# Visualizing Genetics: An Investigation of Dermoscopy as a Tool for Genetic Variant Prediction in Capillary Malformations

**DOI:** 10.1111/pde.70036

**Published:** 2025-10-08

**Authors:** Aretha On, Marie-Chantal Caussade, Allison Britt, Sarah E. Sheppard, Denise Adams, Griffin Stockton Hogrogian, James R. Treat

**Affiliations:** 1 Perelman School of Medicine at the University of Pennsylvania, Philadelphia, Pennsylvania, USA; 2 Section of Dermatology, Children’s Hospital of Philadelphia, Philadelphia, Pennsylvania, USA; 3 Comprehensive Vascular Anomalies Program, Division of Oncology, Children’s Hospital of Philadelphia, Philadelphia, Pennsylvania, USA; 4 Unit on Vascular Malformations, Division of Intramural Research, Eunice Kennedy Shriver National Institute of Child Health and Human Development, Bethesda, Maryland, USA

**Keywords:** capillary malformation, dermoscopy, *EPHB4*, *GNA11*, *GNAQ*, *PIK3CA*, *PIK3R1*, *RASA1*

## Abstract

**Background/Objectives:**

Capillary malformations (CMs) are congenital malformations of capillaries typically visible as blanchable, pink to brown patches on the skin and/or mucosa. The genetic cause of CMs guides diagnosis, treatment, and recurrence counseling. However, identification may be limited by the availability of samples, the type of tests, and insurance coverage. We hypothesize that there are distinct dermoscopic features associated with specific genotypes of congenital CMs.

**Methods:**

A single-center, retrospective cohort study of 22 patients with CMs affecting the skin, a polarized dermoscopic photo of the lesion, and a single nucleotide variant in the *EPHB4, GNA11/GNAQ, PIK3CA/PIK3R1,* or *RASA1* genes was performed. Three reviewers analyzed dermoscopic photos for the presence of apparent vessels, branching, lacunae, geometric shape formation, zones of dropout, follicle-sparing, vessel and background color, and length and width of vessels when discernable. Features were categorized by genotype.

**Results:**

*EPHB4*-CMs have visible lengthwise and widthwise cross sections of vessels that exhibit branching. *RASA1*-CMs generally present with merely a red/pink/brown hue without visible vessels. *GNA11* or *GNAQ*-CMs generally present with pink coloration and generally only with visible widthwise cross sections of vessels without branching. Geometric *PIK3CA*-CMs exhibit distinct purple lacunae that indicate a lymphatic component, but the reticulated *PIK3CA*-CMs otherwise demonstrate a varied presentation.

**Conclusion:**

Our research identified distinct genotype–phenotype correlations for CMs by dermoscopy. Dermoscopy can narrow the differential diagnosis, guide genetic testing, and aid in the interpretation of variants of uncertain significance (VUS). This study demonstrates that dermoscopy holds promise in aiding genetic diagnosis and ultimately medical management.

## 1 | Introduction

Capillary malformations (CMs) are a subset of slow-flow vascular anomalies that affect 0.1%–2% of newborns [1]. Congenital CMs result from a pathogenic single nucleotide variant (previously known as mutations) that is associated with a greater number of dilated capillaries on the skin and/or mucosa [2]. Cutaneous lesions appear as blanchable pink, red, purple, or brown patches depending on the number and thickness of blood vessels as well as the background amount of melanin [2]. CMs can occur in isolation or with other findings that may suggest an underlying syndrome [2].

Several genes involved in the pathogenesis of non-syndromic and syndromic CMs have been identified [2]. Pathogenic variants occurring within the RAS/MAPK, PI3K/AKT, and related pathways cause a wide spectrum of CMs among other vascular anomalies [3]. Somatic mosaic pathogenic variants cause the majority of CMs, but germline pathogenic variants in *RASA1* and *EPHB4* cause the familial capillary malformation (CM) syndromes, capillary malformation-arteriovenous malformation 1 and 2 (CM-AVM1 and CM-AVM2), respectively [4, 5]. Although both RAS/MAPK pathway genes and PI3K/AKT pathway genes can cause CMs, typically, PI3K/AKT pathway genes are associated with slow-flow vascular malformations, while RAS/MAPK pathway genes cause high-flow vascular malformations and complex lymphatic anomalies [6].

Appropriate diagnosis of the causative gene is essential for prognostic counseling and therapeutic management. Genetic diagnosis is dependent on sample type, genes tested, and sequencing depth. Additionally, insurance coverage may not always be available for genetic testing. Given the complexities that can be associated with genetic testing, there is a need for other methods to differentiate CMs. CMs affect the epidermis and dermis and therefore can be visualized directly with dermoscopy [7]. We hypothesized that different genotypes of CMs would lead to different endothelial cell behavior and vascular patterning, and that dermoscopy could identify distinct phenotypic features associated with pathogenic variants of CMs.

## 2 | Methods

We conducted a single-center retrospective chart review at the Children’s Hospital of Philadelphia (CHOP), with IRB exemption (Supporting Information, methods) through March 2024. The population of interest was a database of patients with a vascular anomaly referred for genetic testing. All individuals with clinical photos and positive genetic test results were pulled and manually filtered by diagnosis (capillary malformations only) and type of photo (dermoscopy). Inclusion criteria were patients with a pediatric dermatologist-diagnosed CM, a pathogenic, likely pathogenic, or variant of uncertain significance (VUS) in a gene known to be associated with vascular anomalies, and a contact polarized dermoscopy (PD) photo using the Dermlite DL4 dermatoscope. All photos were taken on an iPhone 14 Pro camera through HAIKU, Epic’s electronic medical record mobile application. The most prominent vascular area of the CM was photographed. Patients with a blurry/unfocused polarized dermoscopy photo of the CM, negative genetic testing, or a unique genetic cause not seen in the remainder of the cohort were excluded from analysis. For patients that had multiple dermoscopy photos, the most focused polarized dermoscopy photo was selected.

### 2.1 | Data Collection

Demographic variables (sex) and other characteristics (age at time of photo, skin phototype [8], syndromic diagnosis [9], prior treatment with laser, sclerotherapy, surgery, or medical therapy) and genetic test results were recorded. Each dermoscopic photo was analyzed by three independent reviewers for categorical variables (presence of apparent vessels, branching, geometric shape formation, zones of drop out, and follicle sparing in addition to vessel color and background color (predefined color card) [10, 11]). Each dermoscopic photo was analyzed by the three reviewers together for quantitative variables (vessel length and width when discernable). If a previously unlisted feature was observed, it was documented and described (presence of lacunae), and photos were re-reviewed for this feature. Data were recorded in REDCap (Research Electronic Data Capture).

### 2.2 | Data Analysis

A descriptive statistical analysis was performed to evaluate the stated variables. A value of accordance between reviewers (ABR) was calculated for categorical variables, with a score of 0 representing a variable consistently absent and a score of 3 representing a variable consistently present. Median [range] was calculated for quantitative variables. Analysis was performed to determine if there was an association between skin phototype or treatment and dermoscopy. Refer to Supporting Information for detailed methods. A comparative analysis was performed to determine the presence of differences between the dermoscopy phenotypes of the genetic variants. Data analysis was performed using Excel Online (Microsoft n.d.).

## 3 | Results

### 3.1 | Patient Population

We identified 26 patients with a diagnosed CM on the skin and a genetic test confirming the presence of a pathogenic/likely pathogenic/VUS in a gene known to cause vascular anomalies. Four patients were excluded, three patients due to the lack of focused polarized dermoscopy photos, and one patient with a *PTEN* variant since no further conclusions could be drawn based on the limited sample size. Twenty-two patients were included in the final analysis. The six genes were condensed into four categories based on similar phenotype and pathway. *GNAQ* and *GNA11*-CMs were combined, and *PIK3CA* and *PIK3R1*-CMs were combined. There were nine patients with *PIK3CA* or *PIK3R1*-CMs, six patients with *EPHB4*-CMs, four patients with *GNA11* or *GNAQ*-CMs, and three patients with *RASA1*-CMs (Table 1).

### 3.2 | Gene Variant

**FIGURE 1 |:**
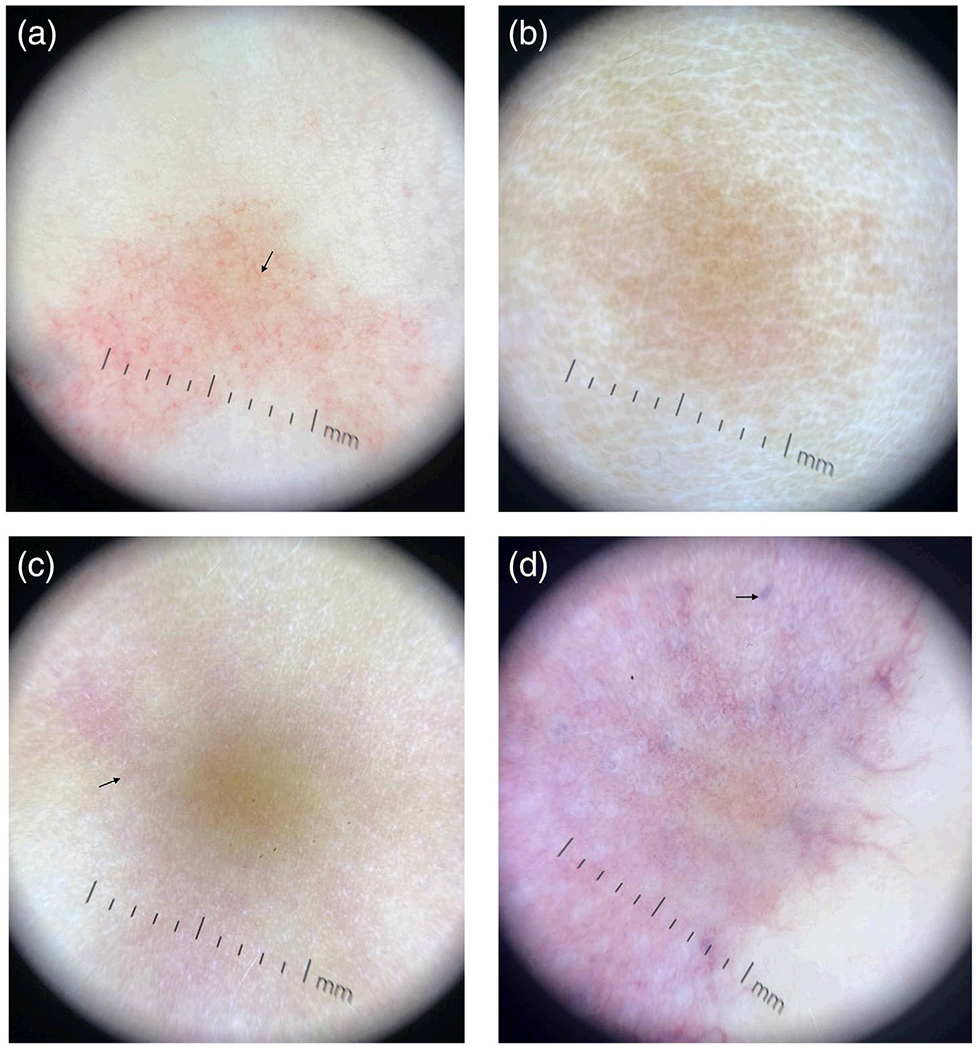
Representative dermoscopy photo for each gene category: (a) *EPHB4*-CM, (b) *RASA1*-CM, (c) *GNA11*-CM, (d) *PIK3CA*-CM. (a) *EPHB4*-CM dermoscopy demonstrating visible lengthwise and widthwise cross sections of vessels that exhibit branching (arrow). (b) *RASA1*-CM dermoscopy demonstrating pink/brown hue without visible vessels. (c) *GNA11*-CM dermoscopy demonstrating pink coloration and no lengthwise cross sections of vessels (arrow). (d) *PIK3CA*-CM dermoscopy demonstrating purple coloration with distinct purple lacunae (arrow).

*EPHB4*-CMs showed a predominantly red vessel color pattern (2.83 ABR), and a pinkish-orange background color (2.50 ABR). *EPHB4*-CMs had visible lengthwise and widthwise sections of vessels (3.00 ABR) that were frequently branching (2.50 ABR) (Figure 1a). The median [range] vessel width was 1.5 mm [1–8], and vessel length was 30 mm [6–360].

*RASA1*-CMs’ color analysis showed pink background color (2.00 ABR) and brown background color (1.00 ABR) (not seen in the dermoscopy of other gene variants), and 2/3 did not present with visible vessels (1.00 ABR) (Figure 1b). The only photo with vessels had width ranging [1–8 mm] and length ranging [11–54 mm]. Due to the lack of visible vessels, ABRs for presence of length (1.00 ABR), width (1.00 ABR), and branching (0.67 ABR) were low.

*GNA11*/*GNAQ*-CMs also showed pink background color (2.75 ABR). *GNA11/GNAQ* showed the highest ABRs for areas of dropout (1.50 ABR) and hair follicle sparing (1.25 ABR). While widthwise sections of vessels were present (3.00 ABR), only 1/4 photos had visible lengthwise sections of vessels (0.75 ABR) (Figure 1c). The vessel width was 2 mm [1–6], and the single photo with vessel length measurements was [7–80 mm].

*PIK3CA*-CMs showed pink (2.44 ABR) and purple (1.67 ABR) background colors and red (1.56 ABR) and purple (1.11 ABR) vessel colors. Additionally, *PIK3CA*- CMs were the only lesions that presented with lacunae (Figure 1d). Of note, evaluation of the morphology of *PIK3CA*-CMs determined six geometric lesions and two reticulated lesions. 5/6 geometric *PIK3CA*-CMs presented with lacunae on dermoscopy, while 0/2 reticulated *PIK3CA*-CMs presented with lacunae. Representative dermoscopy with the associated clinical photo is provided in Supplementary Figure 1. The vessel width was 3 mm [1–27], and vessel length was 20 mm [1–155].

**FIGURE 2 |:**
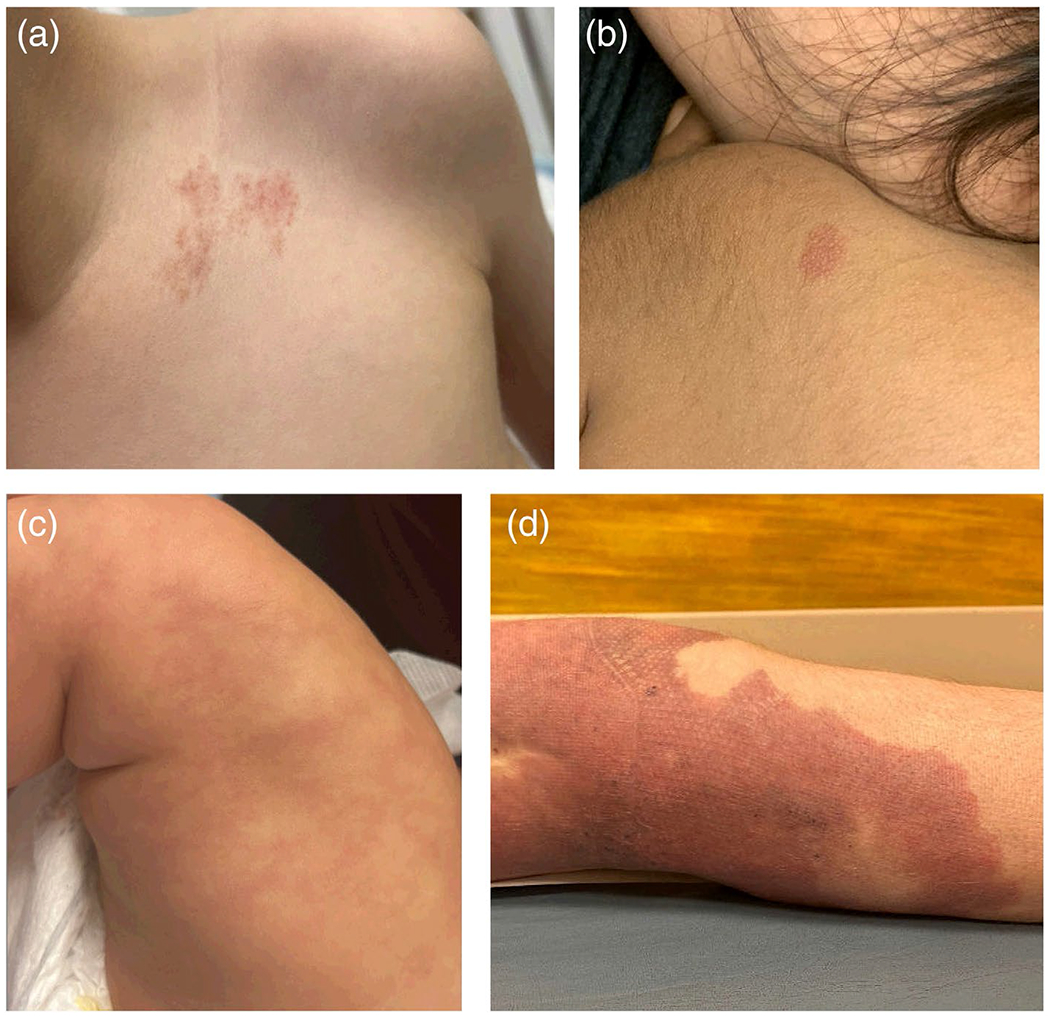
Clinical photos from patients with representative dermoscopy photo for each gene category: (a) *EPHB4*-CM, (b) *RASA1*-CM, (c) *GNA11*-CM, (d) *PIK3CA*-CM.

ABR scores and vessel measurements provided in Tables 2 and 3 respectively. Clinical photos from patients with representative dermoscopy depicted in Figure 1 are shown in Figure 2. Patient characteristics and gene variant information, along with dermoscopy, are provided in Table S1.

### 3.3 | Treatment

Analysis was performed to examine the relationship between presence/absence of any prior treatment and dermoscopic features. No differences were noted in vessel visibility between the treatment-naive and treatment cohort. Out of the 22 patients, 10 (45.5%) patients received treatment for their CM or associated vascular anomalies. Six patients received pulsed dye laser therapy (ranging from 4 to 8 treatments), one patient received debulking treatment, one received sirolimus and sclerotherapy, one received surgery and sclerotherapy, and one received laser, sclerotherapy, and liposuction. When analyzing the potential effect of treatment on the visibility of the vessels along with their color and background color, no clear differences were seen between the two groups (visible widthwise vessels: 2.25 ABR in treatment-naive cohort versus 2.70 ABR in treatment cohort and visible lengthwise vessels: 2.00 ABR in treatment-naive cohort versus 1.50 ABR in treatment cohort).

### 3.4 | Skin Phototype

Moreover, analysis was performed to examine the relationship between the skin phototype classification (as an approximate measure of the baseline amount of melanin in the skin) and the background color and vessel color. Overall, the background colors beige, orange, brown, salmon, red, and blue were evenly distributed across skin types. Pink-orange was noted more commonly in skin type IV (2.25 ABR) compared to skin type I (0.71 ABR), II (1.78 ABR), and III (1.00 ABR). Pink was more commonly noted in skin types I and IV (2.43 ABR, 2.75 ABR) compared to skin types II and III (1.67 ABR, 2.00 ABR). All colors of vessels appeared to be approximately evenly distributed across skin types. However, of note, skin phototype V and VI were not represented in this cohort.

## 4 | Discussion

This study demonstrates that PD allows for the visualization of different vessel characteristics of CMs and can be a complementary tool to effectively assess the genetic pathogenesis of CMs. Likely, the use of contact dermoscopy made the smaller, more superficial vessels harder to visualize but also negated some of the differences after treatment effects. Our findings provide evidence to support the hypothesis that CMs associated with different genetic causes exhibit distinctive patterns under dermoscopy and suggest the potential of dermoscopy to guide the selection of genetic testing, including genes to be evaluated. Despite the distinguishing features, there is enough overlap to warrant the need for genetic testing to determine the definitive diagnosis.

While genetic testing provides valuable information on the causative pathogenic variant for a given congenital CM, there are times when genetic testing is limited by the result of VUS. In these scenarios, dermoscopy may play a role in providing an additional tool in clinical decision-making. Understanding the genetic variant causing a given CM is essential in determining future management. By performing an exam with dermoscopy to evaluate the CM, valuable information can be gleaned to add to the clinical information gathered.

Alterations in the PI3K/AKT pathway are associated with lymphatic malformations, venous malformations, or combined vascular tissue overgrowth; while alterations in the RAS/MAPK pathway are associated with abnormal remodeling and differentiation of vascular tissue, causing capillary and arteriovenous abnormalities that may suggest CNS or other vascular involvement [3, 12]. CMs may be accompanied by other features in syndromic presentations, depending on the type of cell affected and the time of gestation at which the genetic alteration occurred, emphasizing the importance of the identification of the genetic cause in directing medical care and treatment [3, 4].

Some genotypes studied had distinct phenotypes. We found that *EPHB4*-CMs presented with thinner, red vessels on a pinkish-orange background under PD, while *PIK3CA/PIK3R1*-CMs presented with thicker, purple vessels on a purple and/or blue background. The presence of branching vessels that were still visible with contact dermoscopy is highly suggestive of an *EPHB4*-CM, as it was observed in all six patients with this variant, while the presence of lacunae was highly suggestive of a *PIK3CA/PIK3R1*-CM, as it was the only variant exhibiting this feature. Geometric *PIK3CA*-CMs exhibit distinct purple lacunae that indicate a lymphatic component, but the reticulated *PIK3CA*-CMs otherwise demonstrate a varied presentation. A possible explanation for the lacunae in the *PIK3CA/PIK3R1*-CMs is that they are in the small lymphatic areas that are often associated with *PIK3CA/PIK3R1*-CMs [3]. The fact that the *RASA1*-CM vessels were more easily compressed and less visible on contact dermoscopy than *EPHB4*-CMs may be associated with their different effects on vessel endothelial growth. *EPHB4* and *RASA1* both cause CM-AVM syndrome, but they have some clinical differences [5]. Variants in *RASA1* are described as the genetic cause of CM-AVM1 syndrome, which has been reported to be associated with cardiac manifestations and could have a penetrance of up to 99% [5, 13, 14]. On the other hand, pathogenic variants in *EPHB4* explain the phenotype seen in CM-AVM2 syndrome, which is associated with various skin manifestations such as telangiectasias and angiospastic macules, epistaxis, and penetrance reported around 93% [5, 13, 15]. Additionally, dermoscopy in our study was not reliable enough to differentiate between *GNA11* and *GNAQ*-CMs. This is not entirely surprising given the fact that they are homologous genes [3].

Other dermoscopic evaluations in the context of gene variants for CMs and vascular lesions have been described [7, 16]. In contrast to our findings, where vessels were not visualized in two of the three patients with a *RASA1* variant, other case reports of CMs with this variant described a predominant linear branched vascular pattern with an underlying homogeneous brown background [16, 17], We hypothesize that this difference may be explained by the fact that the photos taken in our study used contact PD, while the other reported cases did not utilize contact PD. It is possible that the pressure generated by the contact PD technique may have caused bleaching of the vessels, as described in previous reports [16, 17]. Moreover, these previous reports observed that compression revealed an underlying light brown network (*n* = 4, skin phototype III) [16], while in the patients described in our study (*n* = 3, skin phototype I and III), a pink-salmon color was seen. With the exception of pink background coloration, colors were relatively evenly distributed between skin types in our study. However, a notable feature aligning with other reports of *RASA1*-CM dermoscopy is the brown coloration seen in our *RASA1*-CM that was not noted in the dermoscopy of other genetic variants.

The retrospective design and small sample size are limitations that may affect the generalizability of the findings described in each of these variants. Moreover, with the small sample size, there is an uneven distribution of skin phototypes, with a lack of darker-skinned patients. Additionally, variability is noted in the most represented type of CM (*PIK3CA*). A larger sample size can allow for comparisons between each gene rather than condensed categories of genes. This condensing of genes may limit the identification of unique features present in separate pathogenic variants of *GNAQ* or *PIK3R1*, but not their close counter-parts *GNA11* or *PIK3CA*, respectively. Another limitation is the fact that the degree of pressure applied within contact PD may affect the dermoscopy photo and, therefore, the generalizability of these findings may be limited to other studies using contact dermoscopy. However, contact dermoscopy was consistently used for each dermoscopy photo included in this study, as obtaining high-quality dermoscopy photos in the pediatric population can present challenges at times. Another limitation is the large range in the age of our patient sample. The dermoscopic phenotypic characteristics may appear differently at different ages since a given CM may have had the time to develop, but likely, our results are most relevant to the evaluation of children since our median age was 5.88 years. Nonetheless, this is the largest study of PD analysis in CMs to date, which allowed us to identify characteristics that associate with specific genotypes.

In summary, our study identifies the dermoscopic phenotypic differences in CMs associated with different genetic variants, which can help narrow the differential diagnosis to a single gene and act as a clinical tool to guide genetic testing. This is essential, as accurate diagnosis of the causative gene in vascular anomalies can be important for prognostic counseling and therapeutic management. Future studies utilizing a prospective study design in a larger cohort of patients should be performed to confirm these findings. Additionally, future directions could further evaluate the effect of age on CM appearance and evaluate the differences between syndromic and non-syndromic *PIK3CA/PIK3CA* genetic variants. Dermoscopy holds promise in aiding the genetic diagnosis of CMs and may play a decisive role in cases with VUS.

## Supplementary Material

Supplemental

Additional supporting information can be found online in the Supporting Information section. Data S1: pde70036-sup-0001-Supinfo.docx.

## Figures and Tables

**TABLE 1 |:** Patient cohort demographics and characteristics.

	Sample (*n* = 22; 100%)
Age at dermoscopy photo, median (range), years	5.88 (0.08–34.08)
Sex, *n* (%)	
Male	10 (45.45%)
Female	12 (54.55%)
Skin phototype, *n* (%)	
I–II	16 (73%)
III–IV	6 (27%)
V–VI	0 (0%)
Gene variant, *n* (%)	
*RASA1*	3 (14%)
*EPHB4*	6 (27%)
*GNA11* or *GNAQ*	4 (18%)
*PIK3CA* or *PIK3R1*	9 (41%)

**TABLE 2 |:** Accordance by reviewer (ABR) scores by gene category for categorical variables.

	*RASA1* (*N* = 3)	*EPHB4* (*N* = 6)	*GNA11/GNAQ* (*N* = 4)	*PIK3CA/PIK3R1* (*N* = 9)
Presence of widthwise vessels	1.00	3.00	3.00	2.33
Presence of lengthwise vessels	1.00	3.00	0.75	1.67
Areas of drop out	1.00	1.00	1.50	1.33
Hair follicle sparring	1.00	0.50	1.25	0.67
Geometric shapes	0.33	0.83	0.00	0.33
Branching	0.67	2.50	0.00	0.78

**TABLE 3 |:** Vessel measurements by gene category.

		*RASA1* (*N* = 3)	*EPHB4* (*N* = 6)	*GNA11/GNAQ* (*N* = 4)	*PIK3CA/PIK3R1* (*N* = 9)
Thickness (mm)	Mean (SD)	N/A	2.1 (1.2)	2.5 (1.9)	5.86 (7.8)
	Median	N/A	1.5	2	3
	Range	N/A	1–8	1–6	1–27
Length (mm)	Mean (SD)	N/A	60.4 (97.8)	N/A	45.8 (57.3)
	Median	N/A	30	N/A	20
	Range	N/A	6–360	N/A	1–155

## Data Availability

Due to privacy and ethical restrictions, individual patient data cannot be made publicly available. However, aggregated or de-identified data may be available upon reasonable request, subject to ethical review and approval. Requests for data access can be directed to the corresponding author.
