# Supplementary material for: Visualizing Genetics: An Investigation of Dermoscopy as a Tool for Genetic Variant Prediction in Capillary Malformations
Source: Pediatr Dermatol. 2025 Oct 8;43(1):49–55. doi: 10.1111/pde.70036 (PMC12828651; doi:10.1111/pde.70036)

**Supplementary Materials:**

**Methods:**

The Institutional Review Board at Children’s Hospital of Philadelphia (CHOP) determined that this study (#24-022139) met exemption criteria per 45 CFR 46.104(d) 4(iii). A waiver of HIPAA authorization per 45 CFR 164.512(i)(2)(ii) was granted for accessing identifiable information from the medical records.

Accordance Between Reviewers (ABR)

For categorical variables (except for presence/absence of vessels), three independent reviewers scored each photo as presence (1) or absence (0) of the feature. The sum of these scores was used for each photo, which resulted in a number ranging between 0 and 3. Subsequently, the score from each dermoscopy photo for each gene category was averaged. The calculated values range between 0 and 3 with values closer to 0 representing a variable that is consistently absent for a given gene and a value closer to 3 representing a variable that is more consistently present for a given gene. These calculated values will represent the unit of measure that we will refer to as accordance between reviewers (ABR). One exception is the categorical variable of presence/absence of discernible vessels, that was determined by the three reviewers together rather than separately. Each photo, therefore, was assigned a score of either 0 (absent) or 3 (present) and the ABR was calculated using the average of the scores from each dermoscopy photo for each gene. For quantitative variables, the median and range were calculated for both the shortest and longest length and thinnest and thickest width of vessels, when they were visible. Analysis based on skin phototype was performed to determine if there was an association between various background and vessel colors with the various defined categories in the skin phototype scale by performing the same average calculation described above. Analysis based on treatment was performed to determine if there was an association between visibility of vessels and previous treatment by performing the same average calculation described above.

**Supplementary Tables**

Supplementary Table 1. Patient Characteristics and Gene Variants

Female (F)

Male (M)

Variant of Uncertain Significance (VUS)

| Patient Number | Sex | Skin Phototype | Gene | Variant (c.p.) | American College of Medical Genetics and Genomics (ACMG) Classification | Variant Allele Frequency (VAF), if applicable |  |
| --- | --- | --- | --- | --- | --- | --- | --- |
| 1 | F | II | *EPHB4* | c.1054C>T p.Arg352* c.924C>G p.Tyr308* | Pathogenic  Pathogenic | Heterozygous  Heterozygous | 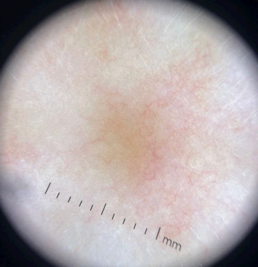 |
| 2 | M | II | *EPHB4* | c.52+1G>C (Splice donor) | Likely pathogenic | Heterozygous | 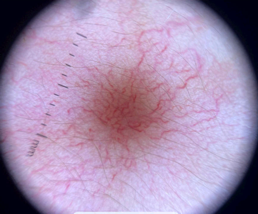 |
| 3 | M | I | *PIK3R1* | c.1690A>G  p.Asn564Asp | pathogenic | 3.3-3.5% in skin | 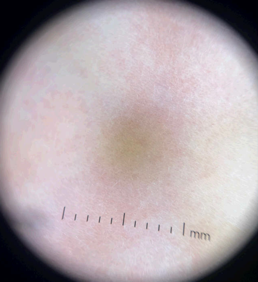 |
| 4 | F | II | *EPHB4* | c.2162T>C p.Leu721Pro | VUS | Heterozygous | 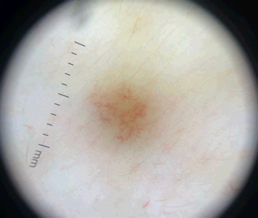 |
| 5 | F | II | *PIK3CA* | c.3019G>C p.Gly1007Arg | Pathogenic | 31% in affected tissue | 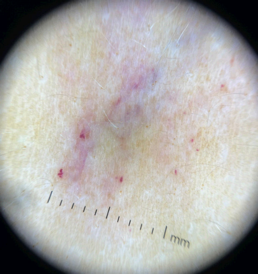 |
| 6 | F | IV | *GNA11* | c.547C>T p.Arg183Cys | Pathogenic | 6.3-6.6% in skin | 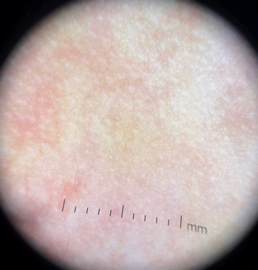 |
| 7 | F | II | *EPHB4* | c.835_836del  p.Leu279Valfs*13 | Pathogenic | Heterozygous | 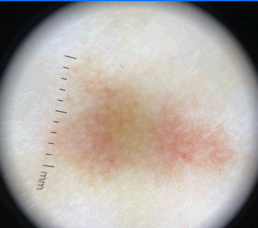 |
| 8 | F | I | *RASA1* | c.2011+1G>A (Splice donor) | Likely Pathogenic | Heterozygous | 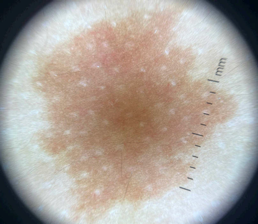 |
| 9 | M | III | *EPHB4* | c.2537C>A p.Pro846His | VUS | Heterozygous | 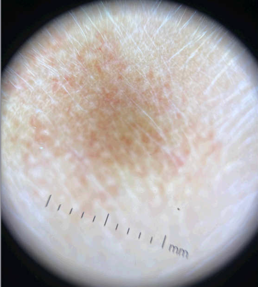 |
| 10 | M | IV | *EPHB4* | c.2484+1G>A (Splice donor) | Likely Pathogenic | Heterozygous | 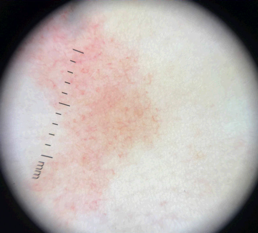 |
| 11 | F | IV | *GNAQ* | c.548G>A p.Arg183Gln | Pathogenic | 2.8-3.5% in skin | 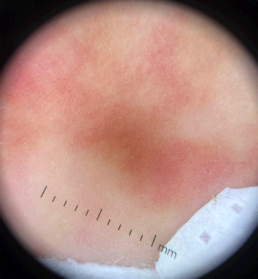 |
| 12 | M | II | *PIK3CA* | c.1031T>G p.Val344Gly  c.646G>A p.Val216Ile | Pathogenic    VUS | 4% in skin    46.8% in blood, 497-50.0% in skin | 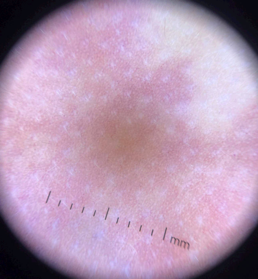 |
| 13 | F | I | *PIK3CA* | c.3125G>A  p.Gly1049Ser | Pathogenic | 6.5% in saliva, 27.9-28.1% in skin | 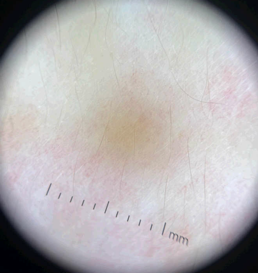 |
| 14 | M | II | *PIK3CA* | c.1633G>A p.Glu545Lys | Pathogenic | 4.7-5.44% in affected tissue | 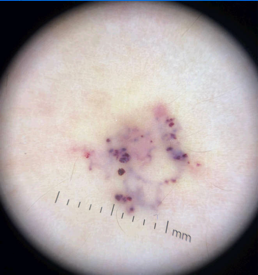 |
| 15 | M | II | *PIK3CA* | c.1624G>A p.Glu542Lys | Pathogenic | 3.3-4.0% in skin | 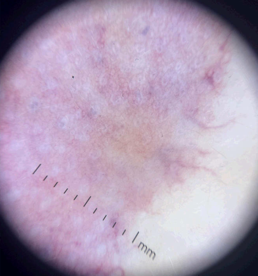 |
| 16 | M | I | *GNA11* | c.547C>T p.Arg183Cys | Pathogenic | 2.5-2.7% in skin | 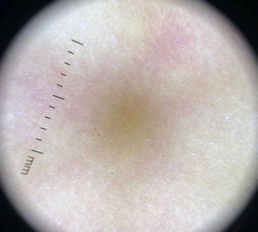 |
| 17 | F | IV | *PIK3CA* | c.1634A>G p.Glu545Gly | Pathogenic | 3.9-4.0% in skin | 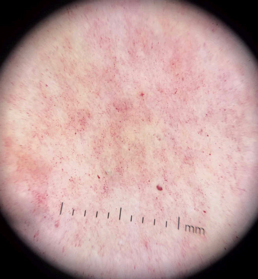 |
| 18 | M | I | *PIK3CA* | c.3012G>A  p.Met1004Ile | Pathogenic | 8.2-8.8% in skin | 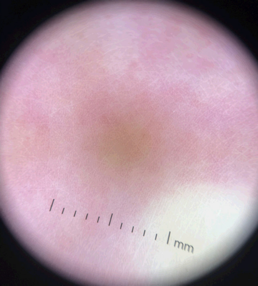 |
| 19 | M | II | *PIK3CA* | c.1634A>G  p.Glu545Gly | Pathogenic | 3.9-4.4% in skin | 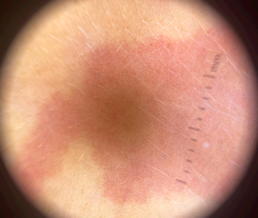 |
| 20 | F | I | *RASA1* | c.625_626delA  p.Ser209* | Pathogenic | Heterozygous | 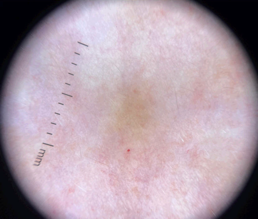 |
| 21 | F | I | *GNA11* | c.547C>T p.Arg183Cys | Pathogenic | 4.58-4.78% in skin | 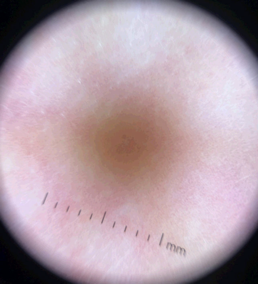 |
| 22 | F | III | *RASA1* | c.2925+1G>T | Pathogenic | Heterozygous | 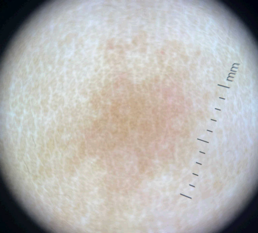 |

**Supplementary Figures**

Supplementary Figure 1. Contrast between geometric *PIK3CA-*CM and reticulated *PIK3CA-*CM. (a) Clinical photo of geometric *PIK3CA*-CM with (b) dermoscopy of the CM demonstrating presence of lacunae. (c) Clinical photo of reticulated *PIK3CA-*CM with (d) dermoscopy of the CM demonstrating lack of lacunae.


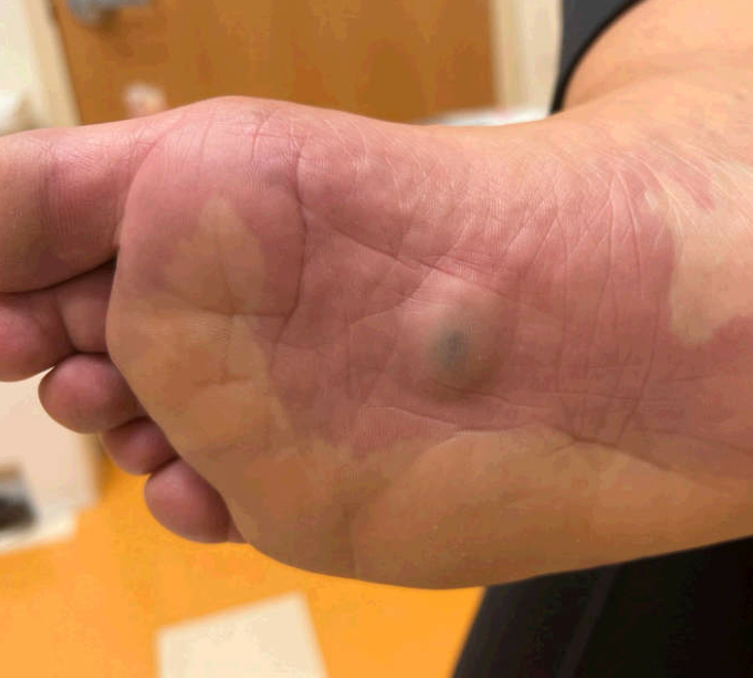

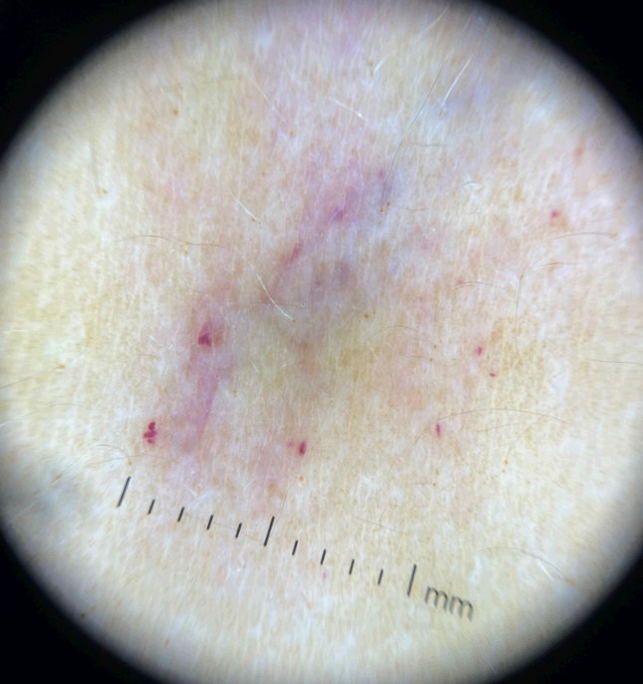

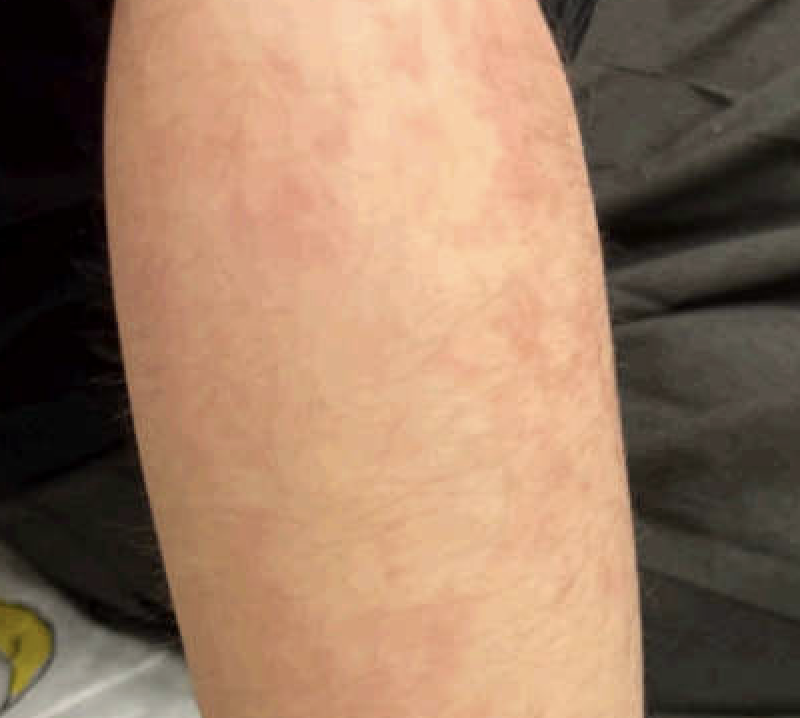

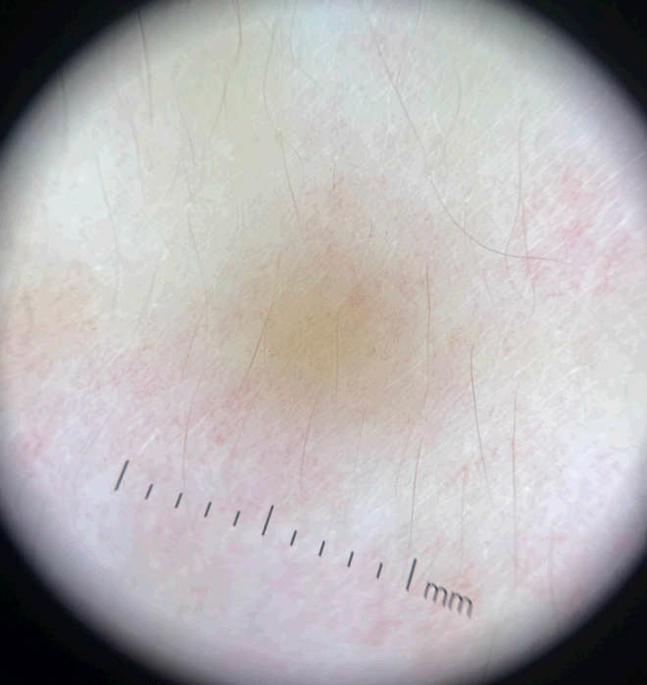

Supplement: Supplementary file 1 — Data S1: pde70036‐sup‐0001‐Supinfo.docx. [file PDE-43-49-s001.docx]
